# Research into the relationship between digital health literacy and healthy lifestyle behaviors: an intergenerational comparison

**DOI:** 10.3389/fpubh.2023.1259412

**Published:** 2023-11-20

**Authors:** Murat Çetin, Rojan Gümüş

**Affiliations:** ^1^Institute of Social Sciences, Dicle University, Diyarbakır, Türkiye; ^2^Atatürk Vocational School of Health Services, Dicle University, Diyarbakır, Türkiye

**Keywords:** digital health, eHealth, health literacy, health information, X Y Z generations, DHLI, healthy lifestyle behavior, Türkiye

## Abstract

**Introduction:**

Digital health literacy wields a pivotal role in individuals’ health status in terms of seeking and choosing appropriate and accurate information, and useful services from a vast array of choices. This study is aimed at assessing the validity and reliability of the Turkish version of Digital Health Literacy Instrument (DHLI) and examining the relationship between DHL and the healthy lifestyle behaviors of participants from X, Y, and Z generations.

**Methods:**

In this study, to conduct a cross-sectional web-based survey, an online self-report questionnaire was built, and a convenience sample with a snowball approach was used. The study was conducted among 1,274 respondents aged between 18 and 64 years. Data collection tools consisted of the Personal Information Form, Lifestyle Behavior Scale II (HLBS II), and DHLI. Cultural validation and psychometric testing of DHLI, exploratory factor analysis, confirmatory factor analysis, Cronbach’s alpha test, and bivariate and multivariate regression analysis were used for statistical analysis.

**Results:**

In the study, the Turkish version of the DHLI tool consisting of six dimensions proved to be valid and reliable, and deemed appropriate for use across all age groups. The average digital health literacy of the respondents was sufficient, but the mean of healthy lifestyle behavior scores was moderate. There was a positive significant relationship between the total mean scores of DHLI and HLBS. Among the subdimensions of DHLI, while the highest mean scores were in DHLI Reliability, DHLI Privacy, and DHLI Search, DHLI Navigation and DHLI Relevance showed the lowest mean scores. DHLI Reliability, DHLI Relevance, and DHLI Adding Content were statistically significant predictors of health-related behaviors of the respondents.

**Conclusion:**

The most important feature and novelty of this study is that, although the DHLI scale has been widely translated for use in many countries, it has been translated and adapted to Turkish for the first time herein. The study offers crucial evidence about Generation X, Y, and Z’s DHL level and its positive relationships with health-related behaviors. Therefore, the community and its partners should lead the way in empowering individuals to understand and use online information in an effective, secure, and health-promoting manner, along with governments.

## 1 Introduction

Advances in technology, the introduction of the Internet into our lives, and the widespread use of computer tablets and smartphones have made access to vast amounts of information both rapid and straightforward. The Internet has become a source of information on almost every subject and is an environment where every person from every nation can access common information, wherever they are in the world (1). As all developments bring both advantages and disadvantages, these innovations have also led to such problems as information pollution and the misleading of consumers. Issues such as gaining access to reliable information and keeping our personal information confidential are also sources of concern that are inherent to this new world (2). According to Al-Turjman et al. developments in information technologies, which are undertaken to increase the harmony between man and machine and facilitate the use of technology, have also enabled many vital applications for people to be transferred to the digital environment (3).

The applications that emerge as part of the rapid progress within the digital world have become an indispensable part of life. Digital applications are used in education, finance, the economy, etc., and vitally, health, one of the most crucial aspects of our lives. WHO declared that the power of digital technologies accelerates global attainment of health and wellbeing (4). Digital transformations in healthcare services push healthcare providers and recipients to seek different ways to make their lives more comfortable (5). One such pursuit is the digitalization of healthcare to reduce the costs associated with health services, allow access to better health information, and provide more effective, efficient, and high-quality health services (6).

People’s access to digital health services has increased with access to the internet and the decrease in the cost of technological devices such as computers, smartphones, and tablets. With the development of digital platforms related to health, individuals have gained the opportunity to perform various activities, such as getting information, receiving services, performing procedures, and shopping in this field. The digital applications and tools that have emerged with opportunities such as technological development and the opening of data infrastructure to the Internet have had a considerable impact on the development of health and health services. Today, patients can get information from hospital web pages for doctor and hospital selection, make online appointments, access test results online, and even be consulted in their homes—without the need to go to the hospital—thanks to telemedicine applications. In addition, they can increase their level of awareness by following healthcare institutions or healthcare professionals on social media (7). These practices and trends also increase competition among healthcare institutions on digital platforms. While consumers access healthcare services digitally, they can also access information on healthy lifestyles such as exercise, healthy diet programs, healthy food, and living habits, on both paid and free platforms. Today, people can also receive personalized exercise or nutrition coaching services for their personal conditions on digital platforms. Warnings and reminders via smart devices are used to ensure that people stick to such programs. In the face of the large amount of information available, people sometimes feel confused and have difficulty accessing appropriate information (8). Therefore, digital health literacy, which combines health literacy and digital literacy, plays an important role in an individual’s life in terms of choosing appropriate information and useful services among the vast array of choices (9).

### 1.1 Health literacy and digital health literacy

Today, people are surrounded by a vast amount of—accurate and inaccurate—information in the digital world, and sufficient health literacy is key to obtaining correct information about health and developing the appropriate health-related behaviors. Health literacy is the degree to which individuals have the skills to search, obtain, comprehend, and use health information and services to promote their health status and that of others (10). The difference between literacy and health literacy is that individuals need additional skills such as the ability to understand and express their complaints and symptoms accurately. Therefore, health literacy plays an important role in promoting the quality of the health services that people utilize (11). According to Parnell et al., good health literacy is essential to understanding medical education leaflets, prescribed medication instructions, and health professionals’ explanations, comprehending consent forms in health institutions, and coping with the complex procedures inherent to the health system (12).

As health literacy is a major factor in the patient-health relationship, the scales measuring patients’ health literacy have been frequently used by various researchers in the literature. Tavousi et al. summarized the most common health literacy instruments in their systematic review (13). The most common health literacy scales encountered in studies investigating health literacy are HALS, REALM, HLSI, and TOFHLA. These scales were validated and adapted in many languages and cultures. The health literacy scales measure individuals’ ability to understand health-related information (13). The studies that used these scales and were conducted in different populations indicate the level of health literacy of the participants, contribute to the quality of health services given, and increase the utilization of health services in an efficient and appropriate manner.

Digital health literacy, or e-health literacy, is defined as seeking, finding, comprehending, and appraising health-related information from electronic sources due to technological developments and addressing and solving any health problem with the information so obtained. Unlike other types of literacy recognized in the literature, DHL combines the six literacy components of computer literacy, traditional literacy, information literacy, health literacy, scientific literacy, and media literacy (14). In the literature, there are different kinds of DHL scales; eHLF, DHLAT, DHLI, and eHeals are the most used by researchers. According to Wang et al. (15), these measurement tools aim to measure digital/e-health literacy and evaluate the degree of health literacy of individuals as being adequate, limited, or inadequate as a result of scoring and measuring individuals’ ability to search, find, understand, evaluate, and use health-related information in digital environments. Validity and reliability studies have been carried out on different samples in different countries, and thanks to these scales the use of e-health services by health service users has been evaluated, and their relationships with certain other variables determined.

### 1.2 Healthy life

Undoubtedly, it is as important to spend our lives in a healthy manner as it is to live long. The way to improve our quality of life is to adopt health-related life habits, which can be defined as behaviors that help human beings to protect and enhance their wellbeing. Healthy nutrition, physical activity, stress management, a good mood, and a fulfilling social life improve quality of life and lead to healthy aging. When we look at the most common chronic diseases in the world, we see cardiovascular diseases such as high blood pressure and heart failure rank among the highest. Just after are joint and bone diseases such as rheumatoid arthritis and osteoarthritis, after which are respiratory infections. Diabetes, neurological disorders, and gastrointestinal diseases are also included in this list. Types of cancer have been increasing continuously and are an important public health issue. These problems, which both individuals and countries are attempting to cope with within complicated health systems, not only affect the individual’s and society’s health but also bring significant long-term financial burdens to both the state and the individual. While the origin of diseases can be traced back to genetic factors, it is also known that they are more likely to be related to living conditions and habits. In addition to environmental factors and stress, the factors that most affect the health of individuals are inappropriate and excessive nutrition, consumption of alcohol, cigarettes, and harmful substances, lack of exercise, and not developing a positive outlook on life. Unhealthy living behaviors negatively affect our physical and mental state and shorten our life spans. When the healthy life behavior development model developed by Walker et al., which has been taken as a reference in many scientific studies, is examined, this model has sub-concepts such as physical activity, nutrition, stress management, interpersonal relations, spiritual growth, health responsibility (16). Walker’s model was used by different researchers. Kuan et al. validated the Walker’s model in the Malay language (17). Bae and Yoon examined health-promoting behaviors among Korean teachers by Walker’s model (18). Holden et al. investigated the relationship between health-promoting behaviors and relationship satisfaction with the same instrument (19). Other Korean researchers Park et al. used Walker’s model and determined lifestyle behaviors in Korean immigrants (20). Although Walker’s model has since been progressed by other researchers, its main sub-concepts have remained the same.

### 1.3 Use of technology in the field of health, e-Health applications

The place of digital technologies and e-health applications in healthcare users’ lives is increasing daily (21). When the 2023 reports on digital technologies were examined, the number of people using the Internet worldwide was 5.16 billion, corresponding to nearly 64.4% of the world’s population. In addition, when we look at world averages, we see that all people spend an average of almost 7 h a day on the Internet. In 2023, it is reported that 95.4% of internet access is via mobile phones, while more than 2 h are spent on social media. The leading reason for the use of the Internet worldwide is to obtain information (57.8%). The number of social media users worldwide is 4.76 billion. Looking at the appropriate statistics for Türkiye, among 85.59 million of the population the total number of Internet users is 71.38 million, and the ratio of this number to that of the entire population is 83.4%. Furthermore, 95.4% of the total population have active cellular phone connection, and 95.4% of people are connected to the Internet via mobile phones. The average time spent on the Internet by the people of Türkiye is close to 8 h per day (22, 23).

Regarding the website languages that generate the most content globally, Turkish ranks 4th after English, Russian, and Spanish. 71% of Türkiye’s population uses social media and spends an average of 3 h a day on it. In Türkiye, the principal reason for people’s use of the internet is that of getting information (88%), followed by news monitoring (70%), brand monitoring (60%), and social connections and friendships (61%). When we look at the health side of the issue, obtaining and searching for health information is among the principal reasons for using the Internet, with a significant rate of 44% (22, 24).

Mainly thanks to online health services, health service users can access all information about the institution from health institutions’ web pages and social media accounts, evaluate alternatives regarding doctors and treatments, and access appointments and test results remotely. By doing online interviews with doctors, they can choose the most appropriate option for themselves while minimizing both financial and moral burdens. In Türkiye, the e-Nabız application by the Ministry of Health is one of the most widespread and easy-to-use applications in Türkiye, which ranked first among the mobile applications installed in 2021 (25). The Life Fits Home (HES) application, used to track health services such as vaccination practices during the COVID-19 pandemic, ranked sixth among all mobile applications regarding the number of active users (26). The total number of e-Nabız users was more than 60 million in 2021, while more than 75 million were using Life Fits Home (HES) (27).

Looking at these statistics, it can be said that the adaptation of Türkiye, with its young and dynamic population, to eHealth applications is extremely high. Turkish people are leading the world in acquiring health-related information and integrating it into their health system. The digital health projects that entered our lives with mobile technologies aim to enable healthcare service users to take on the responsibility for their own health, follow their health status, and provide easy access to healthcare services. In addition, the primary goals of the digital projects are to give patients a central role in managing their diseases, monitoring, and adhering to their medication and treatment, and helping health professionals to identify early signs of deteriorating health before they worsen.

As a result, digital health literacy is crucial for promoting individual health. To the best of our knowledge, most previous studies in Türkiye have used the eHealth Literacy scale developed by Norman and Skinner (28). The eHeals scale can measure individuals’ competence in terms of finding and evaluating health info from online sources via seven questions and one dimension. Although eHealth focuses on seeking and appraising online information, it remains incapable of addressing critical and interactive digital health literacy. Additionally, it does not consider the new tools provided by the internet and technologies (29, 30). On the other hand, other researchers have developed novel measuring tools. One such, DHLI, developed by Van Der Vaart and Drossaert, measures online health information to determine the competence of individuals through seven subdimensions and 21 questions (31). Therefore, it is necessary to verify the validity and reliability of DHLI to use it as a new digital health literacy measuring tool in Türkiye. This study makes valuable contributions because it is the first to have translated and tested the reliability and validity of DHLI in Turkish. Further, DHLI previously saw frequent use among university students or during the pandemic. This research is conducted with various age groups to examine the relationship between DHL (Digital Health Literacy) and HLB (Healthy Lifestyle Behavior).

### 1.4 Digital health literacy and healthy life behaviors

Drawing on the studies in digital/eHealth literacy, a handful of research efforts show that there is a relationship between digital/eHealth literacy and health-related outcomes. Recent studies have shown that high levels of self-reported DHL are associated with better health and more positive health behaviors. On the other hand, low levels of DHL correlate with lower self-care capacity in patients. People with limited or insufficient DHL have difficulties in judging whether they can trust online health information. More recent international studies have demonstrated that inadequate DHL leads to reduced use of health services, reduced ability to make health-related decisions, and poorer health. Further, past studies assert that DHL is associated with access to health services and utilization, higher life expectancy, and decreased healthcare costs (32). According to Kummervold and Wynn (33) and Ekinci et al. (34) individuals with high digital/eHealth literacy have higher competence in terms of searching for and finding appropriate and reliable health information and using health-related applications than people with low digital/eHealth literacy. Moreover, Rosário et al. (35) and Mitsutake et al. (36) found similar results in their studies conducted on different age groups. Additionally, a handful of research efforts have shown that individuals with higher digital/eHealth literacy are more likely to adopt good health behaviors such as healthy food consumption, regular physical exercise (37), and staying away from harmful habits like cigarette and alcohol consumption (38, 39).

Whereas a lot of research has identified an association between digital/eHealth literacy and health-related behaviors there are a limited number of studies conducted across general age groups. Most studies have been conducted with college or university students. In these studies, researchers found that digital/eHealth literacy is an important parameter in promoting healthy lifestyle behavior among college (38), high school (39), and undergraduate students (40). Furthermore, Yang et al. (41) and Balay et al. (42) reported that electronic health literacy is related to health-promoting behaviors. On the other hand, in a limited number of studies examining the relationship between digital/eHealth literacy and healthy life behaviors in the general population, such as healthcare workers (43), and adults (45, 46), researchers found that digital/eHealth literacy promotes healthy life behaviors. Moreover, some research conducted with older groups reported similar results (44, 47). Kim et al., in their meta-analysis research, combined 29 studies that overall suggested a significant relationship between digital health literacy and health–related behaviors, and found a moderate correlation between the two variables (48).

When chronic diseases are considered, previous of research shows that digital health literacy is positively associated with the prevention and management of chronic diseases (49). Higher digital health literacy levels play an important role in the detection and information about cancer in its early stages (50). In a systematic review, Neter and Brainnin examined the correlation between digital/eHealth literacy and healthy life behavior among patients with long-term conditions. They combined 54 studies and found a significant relationship between the two variables. They also determined that there were few studies reporting a relationship between digital/eHealth literacy and health outcomes. They recommended performing studies examining the relationship between digital/eHealth literacy and health outcomes within disadvantaged groups (51).

As digital health literacy became more important during the pandemic and people had difficulties comprehending health information from reliable sources and interpreting them, a lot of studies related to individuals’ digital health literacy have been conducted in the last 3 years. In recent studies conducted during the COVID-19 pandemic in Pakistan (52), South Korea (53), Spain (54), and Hong Kong (55) it was shown that people with higher DHL levels were more competent about actually surviving the disease. In developing/developed countries, internet and technology use of health is spread among all generations mostly among young people. Therefore, most of those studies were conducted with young people as samples. When the points in the first part of this study are considered, digital/eHealth literacy becomes very important in terms of access to reliable and appropriate information that could promote individuals’ health. Summing up, digital health literacy is mandatory to ensure that individuals integrate their knowledge or information into optimal healthy behaviors (52).

### 1.5 Digital health literacy and sociodemographic factors

Past studies evaluating DHL, as carried out on different sociodemographic groups, have revealed that gender, age, marital status, economic status, and education may correlate with DHL levels. In previous research, conducted with adult groups Norman and Skinner (28) and Mitsutake et al. (36) have found that male groups had fewer difficulties in finding digital health information than females. Moreover, studies conducted during COVID-19 period, Rivadeneira et al. (54) and Dadaczynski et al. (56) found higher digital health literacy scores in male groups. On the other hand, Zakar et al. (52), Yuce et al. (57), and Holt et al. (58) revealed that females had higher DHL levels than males. Also, several studies have found no significant correlation between gender and DHL score (37, 59).

Previous research has demonstrated that there is a significant negative correlation between having sufficient levels of DHL and the age of participants. In some studies conducted with different age groups (60, 61), it has been revealed that young adults (Y generation) and middle-aged participants (X generation) had lower digital health literacy than younger individuals (Z generation). Furthermore, in the studies of Jung et al. (62) and Bak et al. (63) it was reported that eHealth literacy was associated with age negatively. Also, the findings of Yuce et al. were in line with those (57). On the other hand, Mitsutake et al. (36) and Xesfingi and Vozikis (37) found a positive association between age and DHL levels. Also, similar findings were reported in studies conducted in Spanish (54) and Dutch (58) populations, stating that the higher the age, the higher the level of eHealth literacy. However, Norman and Skinner (28), Chun et al. (53), and Alipour and Payandeh (59) have pointed out that there is no significant correlation between age and DHL score.

Next, to age, there are also other sociodemographic factors that are associated with participants’ DHL levels, such as economic status, marital status, and education. Past studies have shown that low scores on socioeconomic measures, particularly education and income status, are a handicap for the attainment of DHL. Jung et al., in their review, stated that the higher the level of education, the higher the level of eHealth (62). Also, Mitsutake et al. (36), Xesfingi and Vozikis (37), Rivadeneira et al. (54) and Yuce et al. (57) found similar results. Moreover, a significant positive correlation was found between education and DHL level in some studies conducted with different socioeconomic groups (59, 64). On the other hand, marital status may play a role in DHL score. Xesfingi and Vozikis (37) and Jung et al. (62) reported that married participants had higher DHL.

When the current literature related to digital health literacy and its effect on the health-promoting lifestyle of individuals was reviewed, we determined that there is a lack of new tools measuring digital/eHealth literacy in Turkish. We sought to fill this gap by contributing to the adaption of DHLI into Turkish culture. The purpose of this study is to assess the validity and reliability of the Turkish version of the Digital Health Literacy Instrument (DHLI) and examine the relationship between digital health literacy and the healthy lifestyle behaviors of participants from the X, Y, and Z generations.

## 2 Methods

### 2.1 Study design

A cross-sectional study was performed with data collected in Diyarbakır, Türkiye, in January and February 2023.

### 2.2 Questionnaire design

Data collection tools consisted of three parts. The first part of the questionnaire consisted of a Personal Information Form. The second part was the Lifestyle Behavior Scale II (HLBS II) (65). The last part of the survey was the Digital Health Literacy Instrument (DHLI) (31).

#### 2.2.1 Personal information form

The personal information form consisted of seven Questions involving sociodemographic characteristics. The survey contained questions related to gender, age (Generations X, Y, Z) marital status, income, education, social security status, and participants’ family type. Additionally, a self-administrated questionnaire including six items measuring the health-related behavior of participants as physical exercise, cigarette smoking, fast food consumption, having a chronic disease, weight (kg), and height (cm) was requested. Body Mass Index (BMI) was calculated by the data given by respondents.

#### 2.2.2 Healthy Lifestyle Behavior Scale II

The second part of the survey was the Healthy Lifestyle Behavior Scale II (HLBS II). HLBS II was used to measure the health-related behavior of the participants. HLBS was first developed by Walker et al. in 1987, and subsequently updated by Walker in 1997 and renamed HLBS II. The Turkish validity and reliability study for HLBS II was performed by Bahar et al. (65). Permission was gained from the authors to use this questionnaire. HLBS II is a current and reliable scale used by many researchers (66–70). The HLBS II is a 52-item questionnaire composed of six subdimensions including physical activity, nutrition, stress management, interpersonal relations, spiritual growth, and health responsibility. The items used in the questionnaire rated the health-related behavior of the participants using a four-point Likert scale (never, sometimes, often, and always). Exploratory Factor Analysis (EFA) and Confirmatory Factor Analysis (CFA) were used to test the construct validity of the questionnaire for the current study. The reliability of HLBS was evaluated using Cronbach’s alpha. Good fit index values were obtained from the EFA and CFA results and a six-dimensional questionnaire was yielded for the Healthy Lifestyle Behavior Scale II (HLBS II).

#### 2.2.3 Digital Health Literacy Instrument

The third part of the survey consisted of the Digital Health Literacy Instrument (DHLI) developed by Van Der Vaart and Drossaert, which has been used in various countries and cultures, though not Türkiye. DHLI was first translated and adapted to Turkish and then psychometrically tested in this study. The original version of DHLI was designed for the general Dutch population with a mean age of 46.4 years by van der Vaart and Drossaert, but most of the studies used DHLI to measure digital health literacy of respondents were performed with young groups, especially with university students (40, 52–56).

Additionally, although the original DHLI consisted of seven subscales, in the studies conducted during the COVID-19 period DHLI was validated with five dimensions excluding” operational skills” and “navigational skills” because they were performed with young groups. Dadacynzki et al. who validated the instrument with five scales reported that the university students had the ability of principal technological competence (56). The instrument was used with five subscales by various researchers during the COVID-19 period (40, 52–54). On the other hand, some researchers used three (32) and four (30, 35, 63, 71) subscales to provide the reliability and consistency of the scale in their field studies regarding the digital health literacy level of participants from different sociodemographic groups.

In our study the first subscale “Operational Skills” consists of the questions “How easy or difficult is it for you to a. use the keyboard of a computer (e.g., to type words)?; b. use the mouse (e.g., to put the cursor in the right field or to click)?; c. use the buttons or links and hyperlinks on websites?” was extracted, because these questions were asked to respondents who answered the survey before sending them. Additionally, the respondents were asked if they use social media, have social media accounts, and use health applications such as eNabız, HES, or MHRS at the beginning of the survey. On the other hand, we did not exclude “navigation skills” because our sample consisted of respondents from Y and X generations, therefore the questionnaire included six subscales (Evaluating Reliability, Determining Relevance, Protecting Privacy, Adding Self-Generated Content, Navigation Skills, and Information Searching).

#### 2.2.4 Cultural validation and psychometric testing of DHLI

The DHLI questionnaire developed by Van Der Vaart and Drossaert originally contained seven scales and 21 items. Although it has seven scales (Operational Skills, Evaluating Reliability, Determining Relevance, Protecting Privacy, Adding Self-Generated Content, Navigation Skills, and Information Searching), it was validated and adapted to different cultures and languages various times during the Covid-19 period with five scales (excluding Operational Skills and Adding Content). In this study, as the proportion of Internet users in Türkiye is high, at the beginning of the questionnaire respondents were asked if they were active Internet users, and whether they used a telephone, computer, or tablet to access the Internet. They were asked to continue the questionnaire if the answer was positive. Therefore, the subdimension “operational skills” including basic talents regarding using computers and the Internet was excluded from the questionnaire. The mean score of the remaining six subdimensions of the DHLI was calculated and used for the statistical process. The remaining six subdimensions (DHLI Search, DHLI Content, DHL Relevance, DHL Navigation, DHLI Privacy, and DHLI Reliability) consisted of three to six items on four-point Likert Scales, ranging from “Very easy, rather easy, rather difficult, very difficult” for three subdimensions, and “never, sometimes, often, mostly” for the other three subdimensions, where a higher score indicated higher abilities. The values of the negative statements were reversed to the corresponding positive values.

The first phase in the cross-cultural adaptation of the DHLI into Turkish was to gain permission to do so from Van Der Vaart and Drossaert, who developed the instrument. Secondly, the DHLI questionnaire was translated into Turkish. During the translation and back-translation of the English version of DHLI into the Turkish version the guidelines suggested by Sousa and Rojjanasrirat (72) were followed. The translation of the DHLI, from English to Turkish, was carried out by two professional translators whose native language was Turkish. Both were informed about the content of each item and the constructs of the scale. Also, the study objectives and the target population were explained to them. The back-translation from the Turkish version of the DHLI into English was undertaken by two native English speakers with excellent Turkish language skills. The two versions were compared, and though they were close to each other, minimal discrepancies were solved. Finally, we revised the Turkish version of the DHLI again and sent it to 10 professionals from different fields to check its comprehensibility and practicality. English and Turkish versions of each item were sent online to experts in the fields of health management, public health, nursing, psychology, and computer sciences. They were asked to rate each item’s translation from 1 to 5 (not appropriate to very appropriate). Based on the ratings, suggestions, and comments gained from the experts, the questionnaire was subsequently revised. Except for minor improvements, the experts found each item translation appropriate and practical.

A pilot test was performed to identify and fix any problems with the content, length, and layout of the survey, clarity, and relevance of the questions and the answers. A small sample of respondents was chosen externally (not included in the study again). The pilot tests were applied to the authors’ colleagues in the workplace, their wives/husbands, and their children. The sample of the pilot study was chosen from all socio-demographic groups. With the help of a paper-based, face-to-face survey, any technical issues, errors, or feedback from the respondents were noted by the researchers. Pilot implementation was performed with 20 respondents until no statement was misunderstood. When the Turkish version of the DHLI was field tested cognitively face to face with 20 participants from different sociodemographics, the questionnaire was found to be appropriate and practical.

Finally, the test–retest application of DHLI was performed with 50 volunteers, who after 1 month were asked to answer the questions again. To check the test–retest relation, Intra Correlation Coefficient (ICC) was used. The Turkish version of DHLI and its subscales showed a good test–retest reliability with a total ICC (Intra Correlation Coefficient) of 0.839 (C.I. = 0.717–0.909), and respectively; “DHLI Search” an ICC of 0.805 (CI = 0.656–0.889); “DHLI Content “an ICC of 0.803 (CI = 0.652–0.888); “DHLI Relevance “an ICC of 0.816 (C.I. = 0.677–0.896); “DHLI Navigation “an ICC of 0,734 (C.I. = 0.632–0.849); “DHLI Privacy” an ICC of 0.792 (C.I. = 0.633–0.882) and “DHLI Reliability “an ICC of 0.789 (C.I. = 0.629–0.880). Ultimately, the translation of the DHLI Turkish was completed, and the final version was found to be appropriate for use in the field study.

Exploratory Factor Analysis (EFA) and Confirmatory Factor Analysis (CFA) were used to test the construct validity of the DHLI Turkish version questionnaire. The reliability of the DHLI was evaluated via Cronbach’s alpha internal consistency coefficient. Good fit index values were obtained from the EFA and CFA results, and a six-subdimension questionnaire (Evaluating Reliability, Determining Relevance, Protecting Privacy, Adding Self-Generated Content, Navigation Skills, and Information Searching) was determined.

### 2.3 Data collection and participants

In this study, to conduct a cross-sectional web-based survey, an online self-report questionnaire was built using Google Forms. A convenience sample with a snowball approach was used. An online survey link was distributed across social media, e-mail, and WhatsApp groups, where participation was voluntary. The study was conducted in Diyarbakir, Türkiye, in January–February 2023. The population of the study included people who were living in Diyarbakır and were between 18 and 64 years old. As the proportion of the 65+ age group in Diyarbakır is low (5%) and digital talents are typically low in this group, the 65+ age group was not included in the study (73). Diyarbakır is one of the largest cities in Turkey and different sociodemographic groups live in the city. As in all regions of Turkey Internet and mobile phone usage rate is high in Diyarbakır. The aforementioned rates and statistics were explained in the Introduction section. The inclusion criteria of the sample of the study were being between 18 and 64 years old, living in Diyarbakır province, and having basic computer and Internet skills.

According to official statistical records in Türkiye, 970,764 people were living in Diyarbakır province who were between 18 and 64 years old during the research period (74). Although the sample consisted of 673 people based on a 5% margin of error and a 99% confidence interval, 1,310 participants were included in the study to increase its power. A total of 1,274 questionnaires were found to be valid for data analysis. Participants were divided into three categories according to their age: (1) Generation Z/born 1995–2003, (2) Generation Y/born 1982–1994, and (3) Generation X/born 1965–1981. When an adequate number of participants proportional to age groups and gender was achieved, the online survey part of the study was considered complete.

### 2.4 Ethical considerations

To perform the study, ethical approval was obtained from Dicle University Ethics Committee (E-146791 47–663.05-369714; 05.10.2022/364553). Moreover, permission from the researchers who developed the instruments used and that were validated in the research was gained. Participants were informed about the research and that their personal information would be protected, and data obtained from the study would only be used for the purposes of scientific research. All participants gave informed consent at the beginning of the survey.

### 2.5 Data analysis

In this study, quantitative survey data were analyzed with the Statistical Package for the Social Sciences (SPSS 25.0., Chicago, IL, United States) and Analysis of Moment Structures (AMOS 21, IBM, Armonk, United States) to determine the structural reliability of the model. Regression analysis was used to test the relationship between DHLI and HLBS scores. Descriptive statistics were presented as Mean ± SD, and as frequencies for continuous data, Normality checking showed that the study data were normally distributed. Cronbach’s alpha was used to determine the reliability of the scales. The values of the negative statements were reversed to their corresponding positive values before checking the reliability. Construct validity and reliability were examined using Exploratory Factor Analysis (EFA), the varimax rotation Bartlett’s test, Kaiser-Meier-Olkin (KMO) statistics, and Confirmatory Factor Analysis (CFA). The fit indices used were Chi-Squared/degree of freedom (χ^2^/df), Root mean Residual (RMR), Comparative Fit Index (CFI), Goodness of Fit Index (GFI), Adjusted Goodness of fit index (AGFI), and Root Mean Square Error of Approximation (RMSEA). The model fit was considered suitable for acceptable index values. To find the differences in DHLI and HLBS with respect to sociodemographic characteristics, independent samples Students t-test, One-way ANOVA, and the Scheffe test were used. All analyses with *p* < 0.05 were considered statistically significant.

## 3 Results

### 3.1 Homogeneity, reliability, and descriptive statistical analysis of the subdimensions of the instruments

At the beginning of the study the DHLI and HLBS instruments used in the study were tested, and the homogeneity, reliability, and descriptive statistical analysis of their subdimensions are listed in Table 1.

**Table 1:** Homogeneity, reliability and descriptive statistical analysis of the subdimensions of the instruments.

	Min	Max	Mean	SD	Skewness	Kurtosis	Cronbach
DHLI search	1	4	3.07	0.71	−0.54	0.08	0.907
DHLI content	1	4	2.93	0.74	−0.38	−0.15	0.733
DHLI relevance	1	4	2.77	0.71	−0.07	−0.35	0.892
DHLI navigation	1	4	2.70	0.70	−0.33	−0.24	0.860
DHLI privacy	1	4	3.54	0.70	−1.54	1.57	0.857
DHLI reliability	1	4	3.23	0.76	−0.96	0.56	0.857
DHLI mean	1	4	3.04	0.50	−0.65	1.02	0.896
HLBS physical act	1	4	2.01	0.65	0.79	0.43	0.865
HLBS inter support	1	4	2.92	0.60	−0.14	−0.35	0.866
HLBS health resp	1	4	2.16	0.54	0.83	0.92	0.840
HLBS stress manag	1	4	2.43	0.62	0.48	−0.12	0.823
HLBS spiritual grow	1	4	2.94	0.74	−0.23	−0.78	0.829
HLBS nutrition	1	4	2.35	0.62	0.54	0.43	0.745
HLBS mean	1	4	2.45	0.43	0.31	0.12	0.921

The results of the study showed that the mean score for DHLI was 3.04 with a standard deviation (SD) of 0.50. This means that the average digital health literacy of the respondents was sufficient (over 75%). The HLBS mean score for the participants was 2.45 with a standard deviation (SD) of 0.43. The healthy lifestyle behavior mean score of the respondents was, accordingly, moderate.

The findings in Table 1 indicate that, among the subdimensions of DHLI, the highest mean scores were DHLI Reliability (3.23 ± 0.26), DHLI Privacy (3.54 ± 0.70), and DHLI search (3.07 ± 0.71). DHLI subdimensions showing the lowest mean scores were DHLI Navigation (2.70 ± 0.70) and DHLI Relevance (2.77 ± 0.70). When the mean scores of the subdimensions of HLBS were considered, Spiritual Growth (2.94 ± 0.74) and Interpersonal Support (2.92 ± 0.60) were the highest; on the other hand, Physical Activity (2.01 ± 0.65) and Health Responsibility (2.16 ± 0.54) showed the lowest mean scores. The mean and standard deviation of all dimensions and subdimensions are presented in Table 1.

To examine the homogeneity of the data collected in the research, the Normality test was performed. According to the test results, all sub-dimensions and constructs showed homogeneity. The skewness and kurtosis of the sub-dimensions and constructs of DHLI and HLBS are listed in Table 1.

The reliability of the current study was tested via the Cronbach test. The Cronbach values of DHLI and HLBS total mean scores were 0.896 and 0.921, respectively, which demonstrates good and adequate internal reliability for the overall scale. Additionally, Cronbach’s alpha values for each sub-dimension of the constructs were found to be in acceptable ranges from 0.733 to 0.921 (Table 1).

#### 3.1.1 Exploratory factor analysis results for DHLI and HLBS

Exploratory Factor Analysis (EFA) using principal component analysis with varimax rotation was employed to identify the underlying dimensions of constructs in both DHLI and HLBS II. The Kaiser-Meier Olkin and Bartlett tests were applied before evaluating the results of the exploratory analysis. The only variable with a factor loading greater than 0.50 was extracted. Eigenvalues representing the amount of the total variance explained by the factor that was greater than 1.00 were considered.

All the items had factor loadings of more than 0.50. The factor loadings of subdimensions of DHLI ranged between 0.540 and 0.876 (e.g., the lowest and highest factor loadings are for Search 2 and Privacy 2 subdimensions of DHLI respectively). The KMO was 0.923 and Bartlett’s χ^2^ was 17194.736 (*p* < 0.05). The explained variance for the subdimensions of DHLI were 14.277 for DHLI Reliability, 13.478 for DHLI Relevance, 12.034 for DHLI Privacy, 11.814 for DHLI Content, 8.860 for DHLI Navigation, and 6.859 for DHLI Search. The average variance extracted for the DHLI Turkish version was 67.322. According to the Exploratory Factor Analysis, the DHLI Turkish version is suitable and constitutes a six-subdimension structure.

For HLBS all items had factor loadings of more than 0.50. The factor loadings of subdimensions of HLBS ranged between 0.507 and 0.758 (e.g., the lowest and highest factor loadings are for Nutrition 3 and Physical Activity 2 subdimensions of HLBS respectively). The KMO was 0.938 and Bartlett’s χ^2^ was 26531.987 (*p* < 0.05). The explained variance for the subdimensions of HLBS was 10.113 for Physical Activity, 10.105 for Interpersonal Support, 9.042 for Health Responsibility, 8.254 for Stress Management, 7.878 for Spiritual Growth, and 5.855 for Nutrition. The average variance extracted for HLBS was 51.242. According to the Exploratory Factor Analysis, HLBS is suitable for the current study and constitutes a six-subdimension structure.

#### 3.1.2 Confirmatory factor analysis for DHLI and HLBS

A Confirmatory Factor Analysis was conducted to verify the factor structures of DHLI and HLBS. A summary of goodness of fit statistics for DHLI and HLBS can be found in Table 2.

**Table 2:** Confirmatory factor analysis fit indices for DHLI and HLBS.

Fit indices		Acceptable values	DHLI result	HLBS result
χ^2^		–	493.728	2349.940
df		–	120.000	787.000
χ^2^/df		≤ 5	4.114	2.986
RMR		≤ 0.08	0.025	0.034
GFI		≥ 0.90	0.958	0.930
AGFI		≥ 0.85	0.964	0.902
CFI		≥ 0.90	0.973	0.927
RMSEA		≤ 0.08	0.049	0.039

χ^2^/df, Chi-Squared/degree of freedom; RMR, Root Mean Residual; CFI, Comparative Fit Index; GFI, Goodness of Fit Index; AGFI, Adjusted Goodness of Fit Index; RMSEA, Root Mean Square Error of Approximation.

According to the Confirmatory Factor Analysis, the results for all the fit indices for DHLI were within acceptable ranges. As presented in Table 2, the Chi-Squared/degree of freedom (χ^2^/df = 4.114), Root Mean Residual (RMR = 0.025), Comparative Fit Index (CFI = 0.973), Goodness of Fit Index (GFI = 0.958), Adjusted Goodness of Fit Index (AGFI = 0.964), Root Mean Square Error of Approximation (RMSEA = 0.049) indices all proved to be satisfactory and confirm the unidimensional nature of the model constructs. Based on the results presented in Table 2, all the fit indices for HLBS were also within acceptable ranges. The Chi-Squared/degree of freedom (χ^2^/df = 2.986), Root Mean Residual (RMR = 0.034), Comparative Fit Index (CFI = 0.927), Goodness of Fit Index (GFI = 0.930), Adjusted Goodness of Fit Index (AGFI = 0.902), and Root Mean Square Error of Approximation (RMSEA = 0.039) indices all proved to be satisfactory and confirm the unidimensional of the model constructs. Thus, the CFA results for the measurement model evaluation of DHLI and HLBS ensured that the current model met validity and reliability criteria and that they were suitable for use in further analysis.

### 3.2 Sociodemographic characteristics and health-related behavior of participants

Table 3 shows the sociodemographic information recorded for the participants including gender, age (Generation X, Y, or Z), marital status, income, education, social security status, and family type. Additionally, certain characteristics regarding health-related behavior and status of participants such as physical exercise, cigarette smoking, fast food consumption, chronic disease, and Body Mass Index are listed in Table 3.

**Table 3:** Sociodemographic characteristics and health-related behavior of participants.

		n	%
Sex	Female	611	48.0
	Male	663	52.0
Age	Generation Z (Born in 1995–2003)	461	36.2
	Generation Y (Born in 1982–1994)	402	31.6
	Generation X (Born in 1956–1981)	411	32.3
Marital status	Married	545	42.8
	Unmarried/Divorced/Widowed	729	57.2
Education status	Primary school	64	5.0
	Secondary Education	74	5.8
	High School	186	14.6
	Undergraduate	775	60.8
	Graduate and over	175	13.7
Social security	No	338	26.5
	Yes	936	73.5
Family type	Nuclear family	940	73.8
	Extended family	101	7.9
	Single parent	67	5.3
	Living alone	102	8.0
	Other	64	5.0
Income status	Income < Expenditures	586	46.0
	Income = Expenditures	530	41.6
	Income > Expenditures	158	12.4
BMI (kg/m^2^)	Underweight	116	9.1
	Normal weight	675	53.0
	Overweight	383	30.1
	Obesity class	100	7.8
Cigarettes per day	More than 20	60	4.7
	Nearly 20	146	11.5
	Between 10 and 20	51	4.0
	10	142	11.1
	None	875	68.7
Fast food consumption	Everyday	24	1.9
	4–5 times a week	44	3.5
	2–3 times a week	204	16.0
	Once a week	548	43.0
	Never	454	35.6
Regular physical exercise	Never	373	29.3
	Once a week	423	33.2
	2–3 times a week	350	27.5
	4–5 times a week	80	6.3
	Everyday	48	3.8
Having chronic disease	No	1,035	81.2
	Yes	239	18.8

A total of *n* = 1,310 participants from different sociodemographic groups completed the online questionnaires. Participants (*n* = 36) with systematically missing data were excluded from the study, meaning 1,274 questionnaires were included. Among these 1,274 participants, 611 (48.0%) were female and 663 (52.0%) males.

The number and proportions of respondents from the three generations Z, Y, and X, were 461 (36.2%), 402 (31.6%), and 411 (32.3%), respectively. Most of the study participants were single, at 729 (57.2%). The majority of the respondents (74.0%) were living in a nuclear family and had social security (73.4%). Only 12% of the participants declared that their income was higher than their expenditures. Of all the participants, only 10.8% were educated to below high school degree level. Among the respondents, 37.9% were overweight or obese. Nearly three-quarters of all respondents declared that they did not smoke. The number of participants who had some chronic disease was 239 (18.8%). Moderate fast-food consumption (never/once a week) was very common among respondents (80.0%). Nearly six out of 10 people reported rare (never/once a week) physical activity.

### 3.3 Relationship between participants’ DHLI and HLBS mean scores

In the study, multiple regression and bivariate regression analyses were applied to examine the relationship between the participants’ HLBS and DHLI mean scores and the effect of the subdimensions of DHLI on HLBS. Based on the statistical test analysis, statistically significant results were derived and are presented in Table 4.

**Table 4:** Multivariate regression analysis of DHLI and HLBS mean scores.

Dependent variable	Independent variable	B	SE	*β*	*t*	*p*
HLBS	Const	1.624	0.076		21.685	0.000***
	DHLI search	0.031	0.025	0.050	1.236	0.217
	DHLI content	0.141	0.021	0.241	6.575	0.000***
	DHLI relevance	0.052	0.022	0.085	2.332	0.020*
	DHLI navigation	−0.022	0.017	−0.036	−1.298	0.195
	DHLI privacy	0.009	0.017	0.015	0.558	0.577
	DHLI reliability	0.052	0.021	0.091	2.484	0.013*
	DHLI total^i^	0.308	0.023	0.351	13.379	0.000***

Model *R*^2^ = 0.157; Adjusted *R*^2^ = 0.153; *F* = 39.224; *p* < 0.001; ^i^Model *R*^2^ = 0.123; Adjusted *R*^2^ = 0.125; *F* = 178.988; *p < 0.05; ***p < 0.001.

When the subdimensions of participants’ DHLI mean scores were considered, the multivariate regression analysis showed that the DHLI Content (*β* = 0.241; *p* < 0.001), DHLI Relevance (*β* = 0.085; *p* < 0.05) and DHLI Reliability (*β* = 0.091; *p* < 0.05) subdimensions were statistically significant predictors of HLBS. These variables were found to explain 15.7% of the total variance of the subdimensions of HLBS. According to the bivariate regression analysis, there was a positive, significant relationship between the total DHLI mean score and total HLBS mean score (*β* = 0.351; *p* < 0.001). Furthermore, to examine the relationship between the two variables, a Pearson correlation analysis was performed. According to the test results, there was a positive significantly positive correlation between DHLI total mean score and HLBS total mean score (*r* = 0.351; *p* < 0.001).

### 3.4 Comparison of respondents’ DHLI and HLBS mean scores with respect to sociodemographics and health-related behavior

To explore whether there were differences between the sociodemographic groups in the DHLI and HLBS mean scores, independent t-sample test, One-way ANOVA, and Scheffe tests were performed. Based on the results of the statistical analysis tests, there were significant differences between sociodemographic groups. All statistical tests were two-tailed, and statistical significance was set at 0.05.

To examine the differences in the DHLI and HLBS mean scores based on education, one-way ANOVA and Scheffe tests were performed. The score mean for DHLI (*F* = 109.642; *p* < 0.001) and HLBS (*F* = 5.968; *p* < 0.001) were statistically significantly higher in more educated groups.

Statistically significant differences in DHLI and HLBS mean scores were observed with respect to participants’ economic status. Participants from higher income groups had higher digital health literacy (*F* = 13.778; *p* < 0.001) and health-related behaviors (*F* = 13.295; *p* < 0.001) mean scores.

It was also found that there was a significant difference in generation groups with regard to DHLI and HLBS mean scores. The participants from Generation Y had higher DHLI (*F* = 21.010; *p* < 0.001) and HLBS mean score (*F* = 3.939; *p* < 0.05) than those from Generations X and Z.

Our results indicated that single participants were more likely to have a higher DHLI mean score than those who were married, and this was statistically significant (*t* = −4.650; *p* < 0.001). On the other hand, there were no statistically significant differences between male and female groups with regard to DHLI mean score (*t* = 0.165; *p* > 0.05) and HLBS mean score (*t* = 0.482, *p* > 0.05). Also, participants’ family type did not affect the DHLI (*F* = 2.051; *p* > 0.05) and HLBS (*F* = 0.861; *p* > 0.05) mean scores.

Based on the results of the study, the DHLI mean score was found to be higher for participants who had moderate Body Mass Indexes (*t* = 4.017; *p* < 0.01), had social security (*t* = −5,695; *p* < 0.001), and had no chronic diseases (*t* = 3.770; *p* < 0.001). Similarly, those who had no chronic diseases had significantly higher HLBS (*t* = −5.206; *p* < 0.001) mean scores.

To determine whether there were differences in participants’ DHLI and HLBS mean scores with respect to health-related behavior, one-way ANOVA and Scheffe tests were conducted. The results showed that most health-related behavior positively affected HLBS (*F* = 8.876; *p* < 0.001) and DHLI (*F* = 43.980; *p* < 0.001) mean score of the respondents was the frequency of physical activity. Additionally, participants who consumed less fast food were assigned a higher DHLI mean score than those who consumed more (*F* = 11.759; *p* < 0.001). Smokers and non-smokers could not be assigned statistically different DHLI (*F* = 1.328; *p* > 0.05) and HLBS (*F* = 0.716; *p* > 0.05) mean scores.

### 3.5 Comparison of DHLI and HLBS mean scores of respondents with respect to sociodemographics and health-related behavior in the generation X, Y, and Z groups

To examine the difference in participants’ DHLI and HLBS mean scores with respect to sociodemographics and health-related behavior in Generation X, Y, and Z groups, independent t-sample tests, one-way ANOVA, and Scheffe tests were performed. According to the results of the statistical analysis tests, there were significant differences between sociodemographic groups.

Although there was no significant difference between males and females in the total group mean score, when male and female groups were analyzed for Generations X, Y, and Z separately, the results showed that women had a higher DHLI mean score than men in Generation Z (*t* = 2.302; *p* < 0.05). Additionally, ANOVA test results showed that married participants had higher DHLI mean scores than those who were single in the Generation Y group (*t* = 2.302; *p* < 0.05).

The study found that income and level of education positively predict DHLI and HLBS mean scores across all generation groups. Also, having social security was correlated with a positive role in respondents’ DHLI and HLBS mean scores for all age groups. When the Generation X group is considered, being in a nuclear family had a positive effect on the DHLI mean score that was also statistically significant (*F* = 6.232; *p* < 0.01). Another important finding of the study is related to fast food consumption and cigarette smoking. There was a significant positive relationship between cigarette smoking and the DHLI mean score in the Generation Y (*F* = 3.067; *p* < 0.05) and Z (*F* = 3.655; *p* < 0.05) groups. Furthermore, the participants who were consuming more fast food had higher DHLI mean scores in Generation X (*F* = 5.915; *p* < 0.01) and Y (*F* = 12.683; *p* < 0.001) groups. In all age groups, the respondents who pursued physical activity had higher HLBS and DHLI mean scores. In the Generation X group, participants who had chronic diseases had lower HLBS (*F* = 2.320; *p* < 0.05) and DHLI (*F* = 3.825; *p* < 0.001) mean scores.

To examine the predictors of HLBS in the Generation X, Y, and Z groups, multivariate regression analysis was performed. According to the test analysis, in the Generation Z group, DHLI Content (*β* = 0.258; *p* < 0.001), DHLI Search (*β* = 0.147; *p* < 0.05), and DHLI Reliability (*β* = 0.115; *p* < 0.05) subdimensions positively predict HLBS and were statistically significant. In Generation Y, DHLI Content (*β* = 0.324; *p* < 0.001) and DHLI Reliability (*β* = 0.138; *p* < 0.05) subdimensions had a positive statistical effect on HLBS. In Generation X, only the DHLI Relevance (*β* = 0.220; *p* < 0.05) subdimension predicts HLBS positively and is statistically different.

## 4 Discussion

To the best of our knowledge, our study is the first to attempt to adapt DHLI into Turkish. In the study, the Turkish version of the DHLI tool has proven to be valid and reliable. In previous research, DHLI was almost exclusively used for younger groups (38–42). In this study, we examined digital health literacy of different age groups and compared DHL and HLB in Generation X, Y, and Z groups. When the literature was examined, it was found that only one digital health literacy scale (eHealth developed by Norman and Skinner) had been adapted to Turkish and used by various researchers from Türkiye (57, 75–77). As it was generated in only one dimension and seven questions, it may no longer be adequate to measure the DHL of people in detail. On the other hand, the DHLI Turkish version consisted of six subdimensions, namely operational skills, navigation skills, information-seeking skills, evaluating the reliability of the information, evaluating the relevance of information, adding self-generated content to the web, and protecting and respecting privacy. After validation of DHLI participants’ digital health literacy and healthy lifestyle behavior were found related to the sociodemographics of participants. The next step was determining the relationship between DHL and HLB of the participants. Finally, the findings of the study were determined and discussed in terms of X, Y, and Z generations.

### 4.1 Findings of the study related to digital health literacy and healthy lifestyle behaviors of participants

In the current study, DHLI Turkish was found appropriate for use across all age groups and for participants from different sociodemographics. The results of the study showed that the mean score for digital health literacy was 3.04 with a standard deviation (SD) of 0.50. This means that the average digital health literacy of the respondents was sufficient. Although Generations X and Y were included in the study, the results showed that older groups also had sufficient DHLI levels. Türkiye has a very young population and Internet use is widespread within the country. Various health applications are used by citizens. Also, the government sets health applications for people to be integrated into the health system. Therefore, the use of health applications became inevitable.

Among the subdimensions of DHLI, participants scored highest for DHLI Reliability, DHLI Privacy, and DHLI Search. Considering these findings, it can be said that finding the most reliable information and checking different websites to see whether they provide the same information is the most important factor for the participants. Another issue that the respondents consider is protecting the privacy of messages posted on public forums or social media and not sharing their private information. The other factor that the participants stressed was searching for the correct information and using appropriate words to find the information they sought. The findings of the current study are in accordance with the studies of Zakar et al. (52) and Alipour and Payandeh (59).

In this study, DHLI was performed with participants from different generations. The original version of DHLI was designed for the general Dutch population with a mean age of 46.4 years (Van Der Vaart and Grossaert), therefore the implementation of this study is similar to the original research. On the other hand, most of the studies that used DHLI to measure the digital health literacy of respondents were performed with young groups, especially university students (40, 52–54). Additionally, although the original DHLI consisted of seven subscales, in the studies conducted during the COVID-19 period DHLI was applied with five dimensions (56). Furthermore, some researchers validated only three (32) and four (30, 35, 63, 71) subscales of DHLI in their studies. In this study, six subscales of DHLI (Evaluating Reliability, Determining Relevance, Protecting Privacy, Adding Self-Generated Content, Navigation Skills, and Information Searching) were validated and the reliability and consistency of the scale were provided.

When the healthy lifestyle behavior of the participants was considered, the HLBS mean score of the participants in this study was 2.45 with a standard deviation (SD) of 0.43. Based on the outcomes of the study the respondents’ healthy lifestyle behavior score was found to be moderate. When the mean scores of the subdimensions of HLBS are considered, while the scores of Spiritual Growth and Interpersonal Support had the highest mean, Physical Activity and Health Responsibility had the lowest. These findings were compatible with studies of Daşıkan (68) and Akpınar et al. (69) performed in Turkey. Based on the findings of the current research, participants attach less importance to physically healthy life habits and take less responsibility for their own health. These findings were also compatible with the studies of Daşıkan (68) and Akpınar et al. (69). According to the findings of the study relationships with family, friends, and other close acquaintances are important to participants and they value spirituality highly.

### 4.2 Participants’ digital health literacy and healthy life style behaviors in relation to sociodemographic data

When the participants’ sociodemographic characteristics are considered, the current study found significant differences in the DHL mean score with respect to the sociodemographics of the respondents. Mitsutake et al. (36) and Leung et al. (44) reported that low scores on socioeconomic measures such as income status are a handicap to the attainment of DHL. Moreover, Zakar et al. (52) and Alipour and Payandeh (59) a positive relationship between income status and DHL scores. This study found similar results, as income was a significant factor affecting DHL among all age groups. There was a significant difference between different income groups, with higher digital health literacy mean scores for those from higher, rather than lower income groups.

In the literature, several studies found significant differences between male and female groups in terms of DHL levels. While Norman and Skinner (28), Mitsutake et al. (36), Rivadeneira et al. (54), and Dadaczynski et al. (56) reported higher DHL levels in male groups, some others Zakar et al. (52), Yuce et al. (57), and Holt et al. (58) found that female groups were more talented in terms of digital health literacy. In the current study, there was no significant difference in general male and female groups’ DHL levels. This is in accordance with the studies of Xesfingi and Vozikis (37), Alipour and Payandeh (59), and Chun et al. (53).

In this study, participants from Generation Y had higher DHLI and HLBS scores than Generations X and Z. This is in agreement with the studies of Xesfingi and Vozikis (37), Jung et al. (62), and Yuce et al. (57), which reported higher DHL scores for young adults and middle-aged participants. On the other hand, Mitsutake et al. (36), Holt et al. (58), and Rivadeneria et al. (54) found that the higher the age, the higher the DHL levels. Additionally, Chun et al. (53), Norman and Skinner (28), and Alipour and Payandeh (59) reported no significant difference in DHL between age groups. The current study shows different results when compared with this research.

While Xesfingi and Vozikis (37) and Jung et al. (62) reported that married individuals had higher DHL, the current study reported that single participants were more likely to have higher DHLI mean scores than married ones. Our study is in disagreement with these studies. When the level of education level of the participants is considered, most of the previous studies found similar results to the current one regarding the association between DHL and education. Xesfingi and Vozikis (37) and Yuce et al. (57) found that education has a significant effect on DHL levels. Jung et al., in their review, declared that the higher the level of education, the higher the level of eHealth. Alipour and Payandeh (59), Rivadeneria et al. (54), Zakar et al. (52), and Okan et al. (64) also found a significant positive relationship between education and DHL levels. The results of the current study are compatible with these findings.

### 4.3 Digital health literacy and its effect on health-promoting behaviors

One of the aims of this study was to examine the relationship between participants’ DHLI and HLBS mean scores. The current study found that the association between the two variables was high and statistically significant. This finding was compatible with several previous studies which reported that high levels of self-reported DHL correlate with better health and more positive health behaviors, while low levels of DHL are associated with poorer levels of self-care capacity among patients. According to the studies conducted among college (38), high school (39), and undergraduate students (40) digital/eHealth literacy is an important parameter in promoting healthy lifestyle behavior. Furthermore, Yang et al. (41) and Balay-Odao et al. (42) reported that electronic health literacy is related to health-promoting behaviors. Additionally, in studies examining the relationship between digital/eHealth literacy and healthy life behaviors in the general population, such as healthcare workers (43), and adults (45, 46), researchers found that digital/eHealth literacy promotes healthy life behaviors. Also, in older groups similar were found (44, 47). Kim et al., in their meta-analysis research, combined 29 studies that overall suggested a significant relationship between digital health literacy and health–related behaviors, and found a moderate correlation between the two variables (48). This study supports the findings of the previous research.

When the effect of subdimensions of DHL on HLBS was considered, the results showed adding content, evaluating the reliability of the information, and determining relevance are predictors of the HLB of the participants. First, the adding content (DHLI Cont) subdimension of DHLI plays an important role in the healthy behavior score of the participants. In light of these findings, we may say that for the respondents to use social media and health forums appropriately, formulate and express health-related opinions, and feelings in messages is a major factor in having healthy life behavior. Patient participation and being active in health-related decisions was reported as a major factor in previous research (73). Another factor, DHLI Reliability, means evaluating the reliability of information found via digital sources was affecting the participants’ HLB. To find reliable information, decide whether it is written commercial and check different sources to ensure the reliability of the information plays an important role in individuals’ health behaviors. This is in agreement with previous research (78, 79).

The other subdimension, DHLI Relevance, also has a significant effect on HLB of the respondents. To determine whether the information found was applicable to the participants and to use the information about nutrition, medication, health services, etc., in their daily lives was another important factor in the respondents’ HLB level. On the other hand, when the different generation groups were considered, DHLI Cont and DHLI Reliability were also major factors affecting HLB for Generation Z, but DHLI Search was added to Generation Z to predict their HLB.

For participants from Generation Y, DHLI Content and DHLI Reliability were the most predictive subdimensions for HLB as well, but when Generation X was considered among the subdimensions, only DHLI Relevance predicted the participants’ HLB. This might show that older respondents assign importance to the knowledge or health information in the digital environment applicable to them. The findings of the study are in agreement with the study of Jung et al. (62).

### 4.4 Principal findings of the study in generation X, Y, and Z groups

In this study, a further attempt was made to explore the difference between the Generation X, Y, and Z groups’ DHLI and HLBS mean scores based on the respondents’ sociodemographics. While some previous research has demonstrated that there is a significant negative correlation between having sufficient levels of DHL and the age of the participants, others have found a positive association between age and DHL levels. In studies by Yuce et al. (57), Guo et al. (55), and Paige et al. (61), it was revealed that young adults and middle-aged participants have higher eHealth scores. Also, Marzo et al. (71) and Bíró et al. (80) found similar results. This finding is in agreement with the results of the current study. On the other hand, our results conflict with Mitsutake et al. (36), Rivandeneira et al. (54), and Holt et al. (58) who found that DHL is higher among older participants.

When comparisons were made between different generation groups, different and interesting results were observed. For example, fast food consumers in Generation X and Generation Y had higher DHLI mean scores. This result may be interpreted as follows: although the proportion of groups such as housewives and retired people is high in the Generation X group, there is also a segment within this group that actively works. The working segment in Generation X must eat out more often (81). Similarly, fast food is more likely to be eaten by the working segment of Generation Y. In both generations, the working segment is more educated and has a higher income. That these people have high DHLI scores is therefore an expected result (82).

Another interesting finding in this study is that while family type does not affect DHLI scores in the general group of participants, in Generation X, those with a nuclear family type have higher DHLI scores. Uneducated and low-income people are more likely to live in extended families than other family types; therefore, access to devices such as smartphones, tablets, and indeed the Internet in general is somewhat uncommon among lower socioeconomic groups. On the other hand, individuals living in nuclear families can be correlated to having a higher level of education and having the facilities to access the Internet and other digital resources, and therefore their digital health literacy is higher than participants living in extended families.

Another notable finding is that while single people in the overall research population have higher DHLI and HLBS scores, only married people in Generation Y have higher DHLI scores. This might be the case because people in Generation Y are generally married and have children and are of an age where health problems typically start to emerge (82). On the other hand, the digital literacy level of this age group is high. We may say that this age group is more concerned about the health of their families and themselves and is relatively more educated than other groups, so their DHLI scores are higher (82).

Another important finding of this study was that when all groups were evaluated, the HLBS and DHLI mean scores did not differ between males and females. On the other hand, in this study in the Generation Z group, young girls had higher DHLI mean scores than young boys. In general, considering that beauty, physical appearance, and aesthetic concerns are at their highest level in the twenties, and this is stimulated by social media, we can say that young girls are researching more on topics such as taking care of their health, exercise and diet, skincare, and aesthetic surgery (83, 84). Therefore, searching and utilizing digital health data improves their digital health literacy. The findings of this study showed that smokers’ DHLI mean score in the Generation Y and Z groups is higher with respect to non-smokers. This result shows that conscious individuals with high DHLI mean scores in Generations Y and Z do not struggle to overcome their smoking addiction and postpone the age at which they finally quit because they are young and healthy (83, 84).

### 4.5 Strengths and limitations

This study has strengths; one of which is the sample size. To the best of our knowledge, our study is the first to attempt to adapt the Digital Health Literacy Instrument (DHLI) into Turkish. In the study, the Turkish version of the DHLI tool has proven to be valid and reliable. Additionally, although DHLI was almost exclusively used for younger groups in this study, we examined the digital health literacy of different age groups and compared DHLI and HLBS in Generation X, Y, and Z groups.

When the literature was examined, it was found that only one digital health literacy scale, eHealth (Norman and Skinner) had been adapted to Turkish and used by various researchers from Türkiye. As it was generated in only one dimension and seven questions, it may no longer be adequate to measure the DHL of people in detail. On the other hand, the DHLI Turkish version consisted of six subdimensions, namely operational skills, navigation skills, information-seeking skills, evaluating the reliability of the information, evaluating the relevance of information, adding self-generated content to the web, and protecting and respecting privacy.

Our study has some limitations that should be acknowledged. Although Diyarbakır is a city with a high population from different sociodemographic groups, future studies including participants from different regions of Turkey need to be conducted. Studies conducted with larger groups from different locations in the country will present stronger outcomes. Another limitation refers to the fact that all information is self-reported. Additionally, the other limitation of the current study is excluding 65+ age group respondents. Although their population is below 5% in the region and they do not have competencies of using the internet and smartphone, they hear, see, and apply the information spreading out on the internet. Last, as social media were used as part of the recruitment strategy, this could have led to the selection bias excluding the number of respondents without access to digital media. It is recommended to apply the same instrument to older adults and other disadvantaged groups.

### 4.6 Implications of the study

This study has important implications for health professionals, individuals, researchers of field studies, public health policymakers, and governments for seeking and supplying accurate health information on the internet. Further, past studies assert that DHL is associated with access to health services and utilization, higher life expectancy, and decreased healthcare costs. As inadequate digital health literacy leads to reduced ability to make appropriate health-related decisions and poorer health, it becomes a major issue in the context of public health. Moreover, our research results and findings may provide implications to improve internet users’ digital health literacy to promote their health.

The findings of this study illustrate the need for public health policies and health promotion strategies focused on strengthening digital health literacy among populations, guaranteeing equity in access to information and in the skills to manage, discriminate, and apply information to health. The study also revealed that people’s privacy and the reliability of information available on the Internet is the most important issue for citizens. The government should strengthen the policy guidance and the reliability and security of health-related information. Also, the community and its partners should lead the way in empowering individuals to understand and use online information in an effective and health-promoting manner.

## 5 Conclusion

This study has revealed the effects of digital health literacy, which is required for healthcare service users and all consumers to access appropriate and accurate information that will enable them to benefit the most from the Internet environment on healthy lifestyle behaviors. In addition, the relationship between digital health literacy and health-related behavior among individuals from different generations was examined. This study’s most important feature and difference is that although the DHLI scale has been widely translated for use in many countries, it has been translated and adapted to Turkish for the first time herein. The results suggest that the Turkish version of the DHLI tool is valid and reliable. As DHLI has previously been conducted mostly with university or college students, there is only a limited number of studies measuring the DHLI of participants from different age groups. The study has provided helpful evidence about Generation X, Y, and Z’s DHLI levels and their relationships with health-related behavior. It is obvious that the greater the DHLI level, the healthier the lifestyle behavior at all ages.

To sum up, based on the results of our study, the DHLI score is highest among people who do not have chronic diseases, do physical exercise, consume less fast food, have a normal BMI index, and have health insurance. According to this result, it can be said that people who can access accurate health information in the digital and Internet environment and use digital health services, digital coaching, exercise, nutrition, or services related to health, briefly have high digital health literacy, pay attention to their diet, exercise more, try to avoid being overweight, and make the effort to gain access to health insurance.

Turkish version of the Digital Health Literacy Instrument’s 6 performance-based items is available in Supplementary Appendix. The questionnaire designed by the authors is available and may be used on request via the corresponding author.

## Data availability statement

The raw data supporting the conclusions of this article will be made available by the authors, without undue reservation.

## Ethics statement

The studies involving humans were approved by the Dicle University Ethics Committee. The studies were conducted in accordance with the local legislation and institutional requirements. The participants provided their written informed consent to participate in this study.

## Author contributions

RG: Conceptualization, Data curation, Formal analysis, Investigation, Methodology, Software, Supervision, Validation, Visualization, Writing – original draft, Writing – review & editing. MÇ: Data curation, Investigation, Visualization, Writing – original draft, Writing – review & editing.
