# Supplementary material for: Research into the relationship between digital health literacy and healthy lifestyle behaviors: an intergenerational comparison
Source: Front Public Health. 2023 Nov 20;11:1259412. doi: 10.3389/fpubh.2023.1259412 (PMC10699138; doi:10.3389/fpubh.2023.1259412)
Supplement: Supplementary file 1 [file Data_Sheet_1.docx]

Appendix A. English and Turkish Versions of Digital Health Literacy Instrument

| **DIGITAL HEALTH LITERACY INSTRUMENT (DHLI)** | **DİJİTAL SAĞLIK OKURYAZARLIĞI ÖLÇEĞİ**  **(DSO Ölçeği)** |
| --- | --- |
| **Information Searching** | **Bilgi Arama** |
| When you search the internet for information on health,how easy or difficult is it for you to…  ***(Very easy, Rather easy, Rather difficult, Very difficult)*** | İnternette sağlıkla ilgili bilgi tararken aşağıdaki eylemleri gerçekleştirmek sizin için ne kadar zordur?  ***(Oldukça kolaydır, Kolaydır, Zordur, Oldukça zordur)*** |
| 1…make a choice from all the information you find? | 1. Bulduğunuz tüm bilgiler arasından seçim yapmak ... |
| 2...use the proper words or search query to find the information you are looking for? | 2. Aradığınız bilgiyi bulmak için doğru kelimeleri veya arama sorgusunu kullanmak … |
| 3…find the exact information you are looking for? | 3. Tam olarak aradığınız bilgiyi bulmak … |
| **Evaluating Reliability** | **Güvenilirliğin Değerlendirilmesi** |
| When you search the internet for information on health,  how easy or difficult is it for you to…  ***(Very easy, Rather easy, Rather difficult, Very difficult)*** | İnternette sağlıkla ilgili bilgi tararken aşağıdaki eylemleri gerçekleştirmek sizin için ne kadar zordur?  ***(Oldukça kolaydır, Kolaydır, Zordur, Oldukça zordur)*** |
| 4…decide whether the information is reliable or not? | 4. Bilgilerin güvenilir olup olmadığına karar vermek ... |
| 5…decide whether the information is written with  commercial interests? (e.g. by people trying to sell a  product?)… | 5. Bilgilerin ticari çıkarlarla (örn. ürünü pazarlamaya çalışanlar tarafından) yazılıp yazılmadığına karar vermek ... |
| 6…check different websites to see whether they provide  the same information? | 6. Farklı web sitelerinin aynı bilgiyi sağlayıp sağlamadıklarını kontrol etmek ... |
| **Determining Relevance** | **İlgi Düzeyini Belirleme** |
| When you search the internet for information on health,how easy or difficult is it for you to…  ***(Very easy, Rather easy, Rather difficult, Very difficult)*** | İnternette sağlıkla ilgili bilgi tararken aşağıdaki eylemleri gerçekleştirmek sizin için ne kadar zordur?  ***(Oldukça kolaydır, Kolaydır, Zordur, Oldukça zordur)*** |
| 7...decide if the information you found is applicable to you? | 7. Bulduğunuz bilgilerin sizin için uygulanabilir olup olmadığına karar vermek ... |
| 8...apply the information you found in your daily life? | 8. Bulduğunuz bilgileri günlük yaşamınızda uygulamak ... |
| 9... use the information you found to make decisions about your health (e.g. on nutrition, medication or to decide whether to ask a doctor’s opinion)? | 9. Bulduğunuz bilgileri sağlığınızla ilgili kararlarınızda (örn. beslenme, tedavi veya doktor görüşü alıp almamak gibi) kullanmaya karar vermek… |
| **Navigation Skills** | **Yön Bulma Becerileri** |
| When you search the internet for health information how often does it happen that….  ***(Never, Sometimes, Often, Mostly)*** | İnternette sağlıkla ilgili bilgi taradığınızda aşağıdaki deneyimleri ne sıklıkla yaşıyorsunuz?  ***(Hiçbir zaman, Nadiren, Arada sırada, Sık sık)*** |
| 10…you lose track of where you are on a website or the internet? | 10. Bir web sitesinde veya internette nerede olduğunuzun izini kaybeder misiniz? |
| 11...you do not know how to return to a previous page? | 11. Bir önceki sayfaya nasıl döneceğinizi bilemediğiniz olur mu? |
| 12… you click on something and get to see something different than you expected? | 12. Bir şeye tıklayıp beklediğinizden farklı bir şey gördüğünüz olur mu? |
| **Adding Content** | **İçerik Ekleme** |
| When typing a message (e.g. to a doctor, on a forum or on social media such as Facebook or Twitter) how easy or difficult is it for you to…  ***(Very easy,Rather easy,Rather difficult, Very difficult)*** | Bir doktora, Facebook, Twitter gibi bir sosyal medya mecrasında veya bir forumda bir mesaj yazarken aşağıdaki eylemleri gerçekleştirmek sizin için ne kadar zordur?  ***(Oldukça kolaydır, Kolaydır, Zordur, Oldukça zordur)*** |
| 13…clearly formulate your question or health-related worry? | 13. Sorunuzu veya sağlıkla ilgili endişenizi açık ve net bir şekilde dile getirmek ... |
| 14… express your opinion, thoughts or feelings in writing? | 14. Görüş, düşünce veya duygularınızı yazılı olarak ifade etmek ... |
| 15… write your message as such, for people to understand exactly what you mean? | 15. İnsanlara mesajınızı tam olarak ne demek istediğinizi anlayacakları şekilde yazmak… |
| **Protecting privacy** | **Gizliliğin korunması** |
| When you post a message on a public forum or social media how often…  ***(Never, Sometimes, Often, Mostly)*** | Herkese açık bir forumda veya sosyal medyada bir mesaj yayınladığınızda aşağıdaki deneyimleri ne sıklıkla yaşıyorsunuz?  ***(Hiçbir zaman, Nadiren, Arada sırada, Sık sık)*** |
| 16…do you find it difficult to judge who can read along? | 16. Mesajınızı kimlerin okuyabileceğini belirlemekte zorlanır mısınız? |
| 17… do you (intentionally or unintentionally) share your own private information (e.g. name or address)? | 17. Mesajınızda kasıtlı veya kasıtsız kendi özel bilgilerinizi (adınız, telefon numaranız, adresiniz gibi) paylaşır mısınız? |
| 18…do you (intentionally or unintentionally) share some else’s private information? | 18. Mesajınızda kasıtlı veya kasıtsız bir başkasının özel bilgilerini (adınız, telefon numaranız, adresiniz gibi) paylaşır mısınız? |
